# Dietary fatty acid quality affects systemic parameters and promotes prostatitis and pre-neoplastic lesions

**DOI:** 10.1038/s41598-019-55882-5

**Published:** 2019-12-17

**Authors:** Danilo Ferrucci, Silas Pinto Silva, Andr Rocha, Lucas Nascimento, André Schwambach Vieira, Sebastião Roberto Taboga, Marcelo Mori, Carlos Lenz-Cesar, Hernandes F. Carvalho

**Affiliations:** 1 Department of Structural and Functional Biology, State University of Campinas, Campinas, SP Brazil; 2 Department of Biochemistry and Tissue Biology, State University of Campinas, State University of Campinas, Campinas, SP Brazil; 3 Department of Biophysics, Federal University of São Paulo, São Paulo, SP Brazil; 4 Institute of Biosciences (IBILCE), São Paulo State University, São José do Rio Preto, SP Brazil; 5 Department of Physics, Sciences Center, Federal University of Ceará, Fortaleza, CE Brazil; 6 National Institute of Science and Technology on Photonics Applied to Cell Biology (INFABiC), Campinas, SP Brazil

**Keywords:** Cellular imaging, Urogenital reproductive disorders

## Abstract

Environmental and nutritional factors, including fatty acids (FA), are associated with prostatitis, benign prostate hyperplasia and prostate cancer. We hypothesized that different FA in normolipidic diets (7%) affect prostate physiology, increasing the susceptibility to prostate disorders. Thus, we fed male C57/BL6 mice with normolipidic diets based on linseed oil, soybean oil or lard (varying saturated and unsaturated FA contents and ω-3/ω-6 ratios) for 12 or 32 weeks after weaning and examined structural and functional parameters of the ventral prostate (VP) in the systemic metabolic context. Mongolian gerbils were included because they present a metabolic detour for low water consumption (i.e., oxidize FA to produce metabolic water). A linseed oil-based diet (LO, 67.4% PUFAs, ω-3/ω-6 = 3.70) resulted in a thermogenic profile, while a soybean oil-based diet (SO, 52.7% PUFAs, ω-3/ω-6 = 0.11) increased body growth and adiposity. Mice fed lard (PF, 13.1% PUFA, ω-3/ω-6 = 0.07) depicted a biphasic growth, resulting in decreased adiposity in adulthood. SO and PF resulted in hepatic steatosis and steatohepatitis, respectively. PF and SO increased prostate epithelial volume, and lard resulted in epithelial hyperplasia. Animals in the LO group had smaller prostates with predominant atrophic epithelia and inflammatory loci. Inflammatory cells were frequent in the VP of PF mice (predominantly stromal) and LO mice (predominantly luminal). RNAseq after 12 weeks revealed good predictors of a later-onset inflammation. The transcriptome unveiled ontologies related to ER stress after 32 weeks on PF diets. In conclusion, different FA qualities result in different metabolic phenotypes and differentially impact prostate size, epithelial volume, inflammation and gene expression.

## Introduction

Benign prostate hyperplasia (BPH) and prostate cancer (PCa) are frequent diseases of the prostate. Despite showing common etiologies, such as androgen dependency and metabolic disorders, BPH and PCa show different symptoms, histopathology and malignity. BPH has been posed as a predisposing condition for PCa development, but this sequence is frequently questioned^1,2^. Importantly, PCa will represent 27% of all new cases of cancer in Brazil and in the USA^3,4^. Prostatitis is also a condition of the prostate gland and is frequently part of the low urinary tract symptoms.

The mechanisms leading to prostatitis, BPH and PCa are not fully elucidated and are certainly multifactorial, influenced by hormones, genetic and inherited factors^5^, ethnicity^6^ and environmental/nutritional exposure^7^. Accordingly, there is a 6–50-fold higher prevalence of PCa in the Western countries and in migrating populations settled in eastern countries, reinforcing the notion that environment and life style impact the susceptibly to PCa and its progression^8–11^. Obesity is related to a plethora of comorbidities and includes sub-clinical systemic inflammation and an altered profile of adipokines. Obesity favors PCa incidence and aggressiveness, and the consumption of dietetic fatty acids is a major etiological factor for BPH and PCa^12^. Increased consumption of saturated fatty acids promotes prostate growth in mice^13^. Consequently, the amount of fatty acid in the typical Western-style diet, obesity (increased adiposity) and PCa risk have been associated. A major variable in this scenario is the quality (profile) of the ingested fatty acids present in each diet^14^; however, much attention has been given to high fat diets.

On the other hand, beneficial effects of poly-unsaturated fatty acids (PUFAs) have been demonstrated, including cardioprotective, immunomodulatory and anti-atherosclerotic effects^15–17^. Total and unsaturated fats have been associated with a reduced risk of PCa^18^. PUFAs were also shown to increase neurogenesis in the hypothalamus and, thereby, to control food ingestion and energy expenditure^19^.

ω-6-rich diets have been associated with an increased risk of PCa. ω-6 fatty acids are converted into even-series eicosanoids, such as leukotrienes, prostaglandins, prostacyclins and troboxans, all pro-inflammatory mediators resulting from the metabolism of arachidonic acid [AA, ω-3/ω-6 ratio = 20:4]. Besides stimulating inflammation, AA stimulates cell proliferation^20^. Additionally, *in vitro* and *in vivo* studies have suggested that ω-3-rich PUFAs, specially α-linolenic (LA, 18:2) and its derivatives docosahexaenoic (DHA, 22:6) and eicosatetraenoic (EPA, 20:5), are anti-inflammatory, pro-apoptotic, anti-angiogenic and anti-proliferative^21,22^. Given these effects, ω-3-rich PUFAs would be inversely associated with BPH and PCa. Nonetheless, contradictory results exist^23^. High plasma levels of docosapentaenoic acid (DPA, 22:5) are inversely correlated with the risk of PCa, while EPA/DHA are positively correlated^24^.

The complexity of the effects of fatty acids on different cell types, organs and the susceptibility to disease lies in the fact that fatty acids are substrates for energy production, plastic elements for the synthesis of biological membranes and the substrate of enzymes producing diverse bioactive molecules, including those affecting pro- and anti-inflammatory pathways^25^. Furthermore, body fat might affect prostate physiology and disease progression, given that prostate epithelial cells express adipokine receptors, such as adiponectin receptor 2^26^. It is possible that fatty acids have unique roles leading to prostatitis, BPH and, possibly, to PCa.

Considering the diagnosis of prostate cancer is frequent in older men and that environmental factors are particularly important in PCa incidence, we are interested in finding factors that affect prostate physiology that might increase the susceptibility to carcinogenesis, favoring the events that give rise to and promote cancer.

In a previous work, we demonstrated that normolipidic diets based on common dietary lipid sources affect epithelial proliferation rate and overall prostate growth in rats, which occurred in parallel with variations in circulating testosterone and estrogen levels, as well as in the expression of the androgen receptor and PPARγ in the epithelium^27^. In that study, we reported an antagonistic effect of the diet, either with prevailing saturated fatty acids (pork fat/lard) or with a predominance of poly-unsaturated fatty acids (high ω-3/ω-6). The former stimulated and the latter restricted proliferation and growth of the gland. In the same study, a prevalence of poly-unsaturated fatty acid with low ω-3/ω-6, showed an intermediate effect in prostate growth and epithelial proliferation^27^. That work included the administration of the diets to a single species (rats) and a short exposure to the diets (ten weeks after weaning). Additionally, a recent study has shown that high-fat diets induce prostate epithelial hyperplasia, achieved through proliferation of basal cells and their differentiation into luminal cells^28^.

Therefore, in this study, we wanted to determine whether exposure to normolipidic diets based on linseed oil (LO, 67.4% PUFAs, ω-3/ω-6 = 3.70), soybean oil (SO, 52.7% PUFAs, ω-3/ω-6 = 0.11) (SO) and pork fat/lard (PF, 13.1% PUFA, ω-3/ω-6 = 0.07), affects the structure and physiology of the ventral prostate (VP), increasing its susceptibility to prostate diseases, using two different species (mice and Mongolian gerbils) and a very long diet exposure (up to 32 weeks after weaning). Additionally, we performed a comprehensive analysis of the systemic/metabolic conditions that could affect the prostate gland.

The results presented here show that normolipidic diets containing different fatty acid compositions affect body adiposity, thermogenic capacity and the cytokine/adipokine profile, which reflected on marked changes in the epithelium, different inflammatory patterns (prostatitis, in mice and gerbils) and different epithelial lesions (in gerbils). Gene expression profiles and histological changes demonstrated that the diets (and their fatty acid composition) had distinct effects on the prostate, including an inflammatory signature and endoplasmic reticulum (ER) stress.

## Results

### A diet with predominantly ω-3 poly-unsaturated fatty acids (LO) promotes less weight gain and a better metabolic profile, compared to diets rich in ω-6 polyunsaturated (SO) or saturated fatty acids (PF)

**Figure 1:**
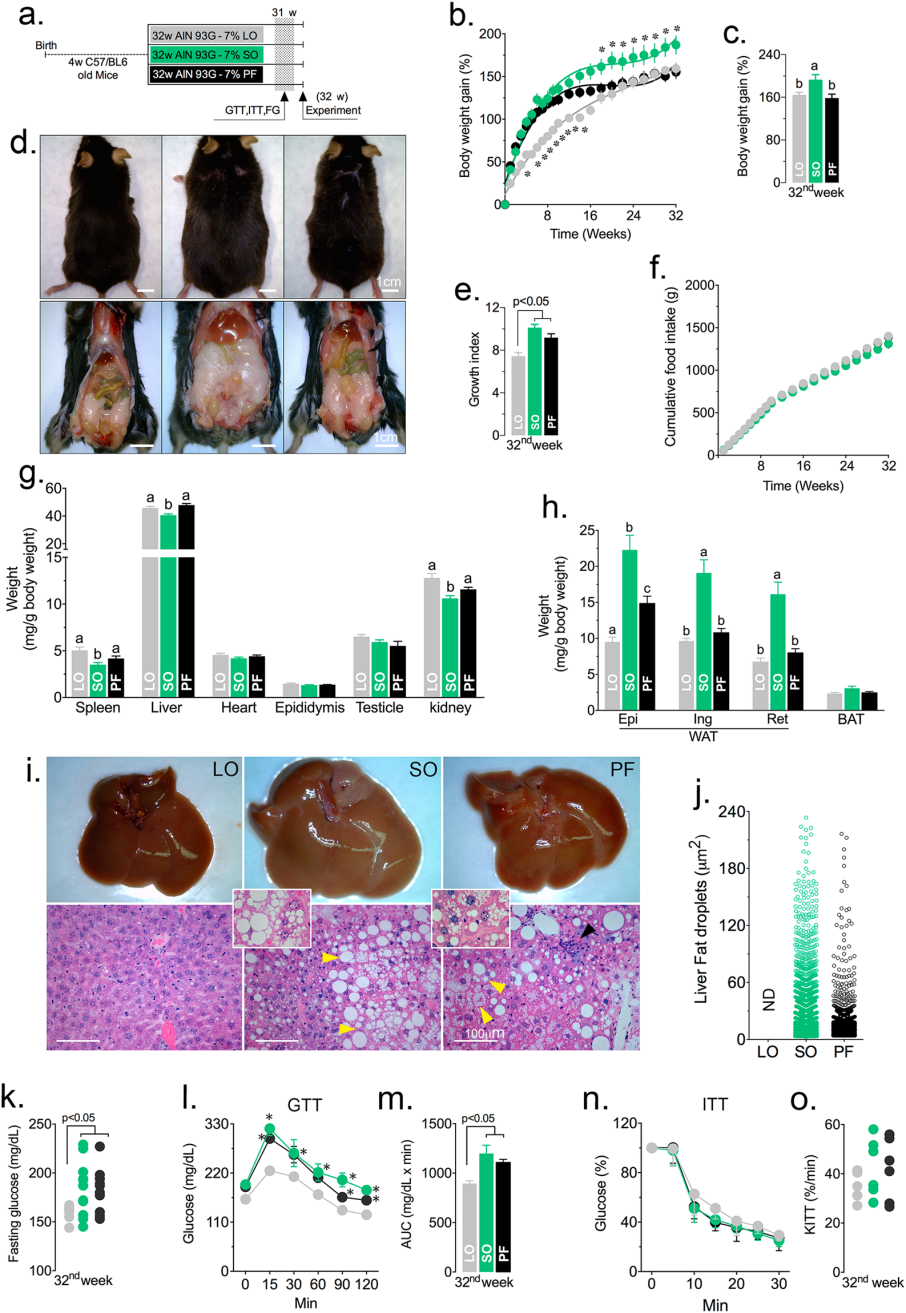
Dietary fatty acids in normolipidic diets affect growth, adiposity, liver and glucose metabolism. C57/BL6 mice were fed normolipidic diets containing different fatty acid compositions for 32 weeks (**a**). Body weight increase (**b**,**c**), growth indices (**d**,**e**) and food consumption (**f**) were determined weekly. After 32 weeks on the specified diets, mice were euthanized and various organs (**g**) and adipose tissues (**h**) were weighed The livers were severely affected on visual inspection and were further analyzed by histology after HE staining, including assessment of lipid droplet content and sizes (**i**,**j**,) N.D. = not-detected. The arrowhead points to an inflammatory focus. After 31 weeks on the specified diets, mice had their fasting glucose concentration measured (**k**) and sequentially submitted to the glucose tolerance test (GTT; **l**, mean glucose concentration; **m**, area under the curve) and the insulin tolerance test (ITT; **n**, mean glucose concentration; **o**, decay constant). n = 12 (**a**–**h**) or n = 6 (**i**–**o**); Statistically significant differences are indicated by different letters or asterisks (*p* < 0.05). ANOVA and Tukey’s test.

C57/BL6 mice were randomly divided into three experimental groups and fed normolipidic diets prepared with linseed oil (LO), soybean oil (SO) or lard (PF), as specified (Table S1). These diets had particular fatty acid compositions: linseed contained ω-3 polyunsaturated fatty acids (ω3/ω6 ratio = 4:1), ω-6 polyunsaturated fatty acids predominated in soybean oil (ω3/ω6 ratio = 1:9) and lard was essentially composed of saturated fatty acids, as determined by gas chromatography (Table S2). The diets were given immediately after weaning for 12 (Fig. S1a) or 32 weeks (Fig. 1a).

After 12 weeks on the specified diets, animals fed SO diets gained more weight than the other groups (Fig. S1b–e), though food intake and total calories were the same (Fig. S1f). Internal organs were similar sizes between groups, except for the liver, which was heavier in the PF group (Fig. S1g). White adipose tissues were larger in mice fed PF, while the brown adipose tissue was smaller (Fig. S1h). Liver histology was normal for the LO group, while there were signs of steatosis and steatohepatitis in the SO and PF groups, respectively (Fig. S1i, j). Fasting glucose, glucose tolerance and insulin tolerance were similar among the three groups (Fig. S1k–o).

Animals fed a diet containing high content of ω-3 (LO) for 32 weeks showed a smaller growth index and less body weight gain (Fig. 1b–f), reduced adiposity (Fig. 1g,h), preserved liver histology (Fig. 1i,j), normal fasting plasma glucose levels (Fig. 1k) and reduced area under the curve in GTT (Fig. 1l,m), suggesting better tolerance to glucose. Insulin tolerance was similar among the three experimental groups (Fig. 1n,o). Mice fed a ω-6 rich diet (SO) had elevated fasting plasma glucose levels (Fig. 1k), impaired glucose tolerance (Fig. 1l,m) and liver steatosis (Fig. 1i,j). Animals fed PF had severe liver steatohepatitis (Fig. 1i). Surprisingly, the diet prepared with SO was more obesogenic than that prepared with PF and resulted in increased growth (Fig. 1d,e), body weight gain (Fig. 1b,c) and adiposity (Fig. 1h).

### An ω-3-rich, normolipidic diet promotes increased metabolism and thermogenesis associated with reduced spontaneous physical activity

**Figure 2:**
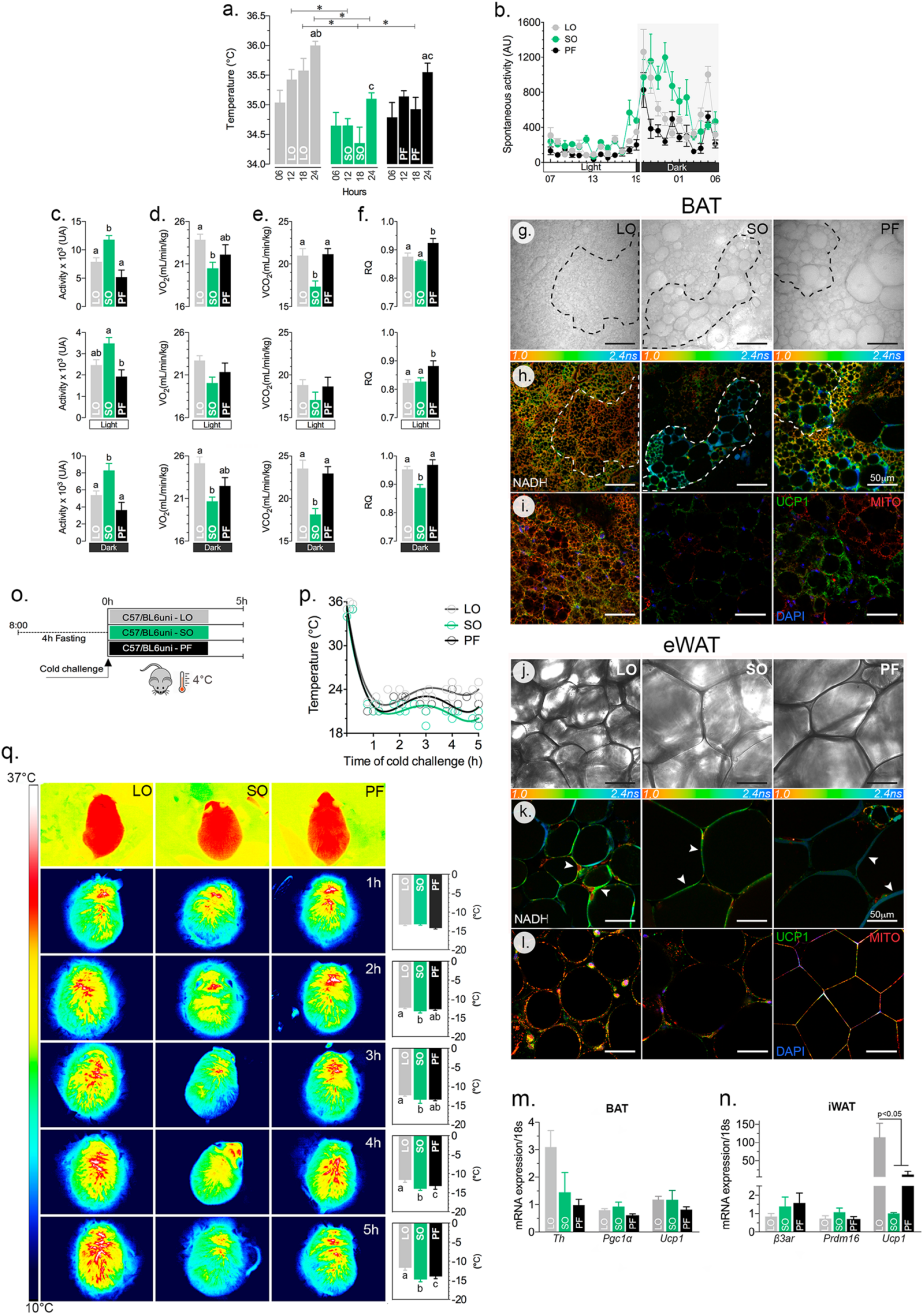
Dietary fatty acids in normolipidic diets affect energy metabolism in the adipose tissues and thermogenesis. C57/BL6 mice fed the specified diets for 32 weeks were evaluated for their body temperature (**a**), spontaneous physical activity (**b**,**c**), O_2_ consumption (**d**), CO_2_ production (**e**) and respiratory ratio (CO_2_ production/O_2_ consumption; **f**) during the light/dark cycles for 24 h at 22 °C. At the end of the 32 weeks, samples from the interscapular brown adipose tissue (BAT; **g**,**h**,**i**) and the epididimal white adipose tissue (eWAT; **j**,**k**,**l**) were collected for the histological analyses, redox state (NADH) using FLIM, mitochondrial content and UCP-1 immunofluorescence (**i**,**l**). Several markers of adipocytes were examined at the mRNA level in BAT (**m**) and eWAT (**n**) and revealed a significant increase in the expression of *Ucp1* in the latter, suggesting browning. One week before the end of the experiment (31^st^ week), the animals were exposed to 4 °C for 5 h to evaluate cold-induced thermogenesis (**o**). The body surface temperature was determined hourly (**p**), using a thermostatic camera (**q**). Dashed lines in panels g and h delimitate areas differing in metabolic activity in the BAT among LO, SO, PF. The arrowheads in panels **i**–**l** indicate regions with high metabolic activity among the two adipose tissue cells; n = 6 (**a**–**f**, **o**–**q**) or n = 3 (**g**–**n**); Statistically significant differences are indicated by letters or asterisks (*p* < 0.05). ANOVA and Tukey’s test.

Additional animals from the three experimental groups were used to investigate the energy metabolism and the thermogenic phenotype. To understand how body weight and adiposity are affected without changes in food intake, we investigated energy metabolism and the thermogenic capacity of mice fed different types of fatty acids. Mice fed the ω-3-rich diet (LO) showed higher body temperature (Fig. 2a), increased O_2_ consumption (Fig. 2d) and CO_2_ production (Fig. 2e) and a smaller respiratory ratio (CO_2_/O_2_, Fig. 2f) at the end of the light/dark cycle. These animals showed intermediate levels of spontaneous physical activity (Fig. 2b,c), compared to those fed SO diets.

After 32 weeks on the specified diets, the interscapular brown adipose tissue (BAT, Fig. 2g) and the epididimal white adipose tissue (eWAT, Fig. 2j) morphology, fluorescence life time imaging microscopy (FLIM), mitochondrial and the UCP1 content were examined. There was a clear preservation of the brown features in the BAT of LO-treated animals, while those in the SO and PF groups showed a mixed phenotype. On the other hand, there was a browning (i.e., multilocular lipid droplets) of the inguinal white adiposte tissue.

Using FLIM, we have identified changes in the lifetime of NADH autofluorescence (Fig. 2h,k) and found an evident extension of the lifetime in the SO and PF adipocytes. Accordingly, the mitochondrial content and UCP1 immunostaining were greater in the LO-treated mice compared to the two other groups (Fig. 2i,l). These results show that the ω-3-based diet (LO) promotes a more thermogenic phenotype in both BAT and eWAT. To confirm this phenotype, we checked the expression of several brown adipocyte markers and found an increase in *Ucp1* expression in the eWAT of animals fed an LO diet compared to those on SO and PF diets (Fig. 2m,n).

To evaluate acute cold-induced thermogenesis, the animals were submitted to 4 °C for 5 h (Fig. 2o). The cold-induced thermogenesis was more effectively controlled by animals in the LO group, though the other groups were also cold-resistant (Fig. 2p). The thermal images acquired and the variation in temperature along the test are presented in Fig. 2q. Curiously, mice fed an ω-6 rich diet (SO) presented a lower body surface temperature (Fig. 2a), decreased energy expenditure (Fig. 2d,e) and, despite showing increased spontaneous physical activity, showed a worse thermogenic response to cold. Surprisingly, the animals fed a normolipidic diet, rich in saturated fatty acids (PF), showed a metabolic and thermogenic phenotype intermediate to the LO and SO.

### A normolipidic, saturated fatty acid-rich diet (PF) increases the serum concentration of pro-inflammatory cytokines, opposing the effects of an ω-3-rich diet (LO)

**Figure 3:**
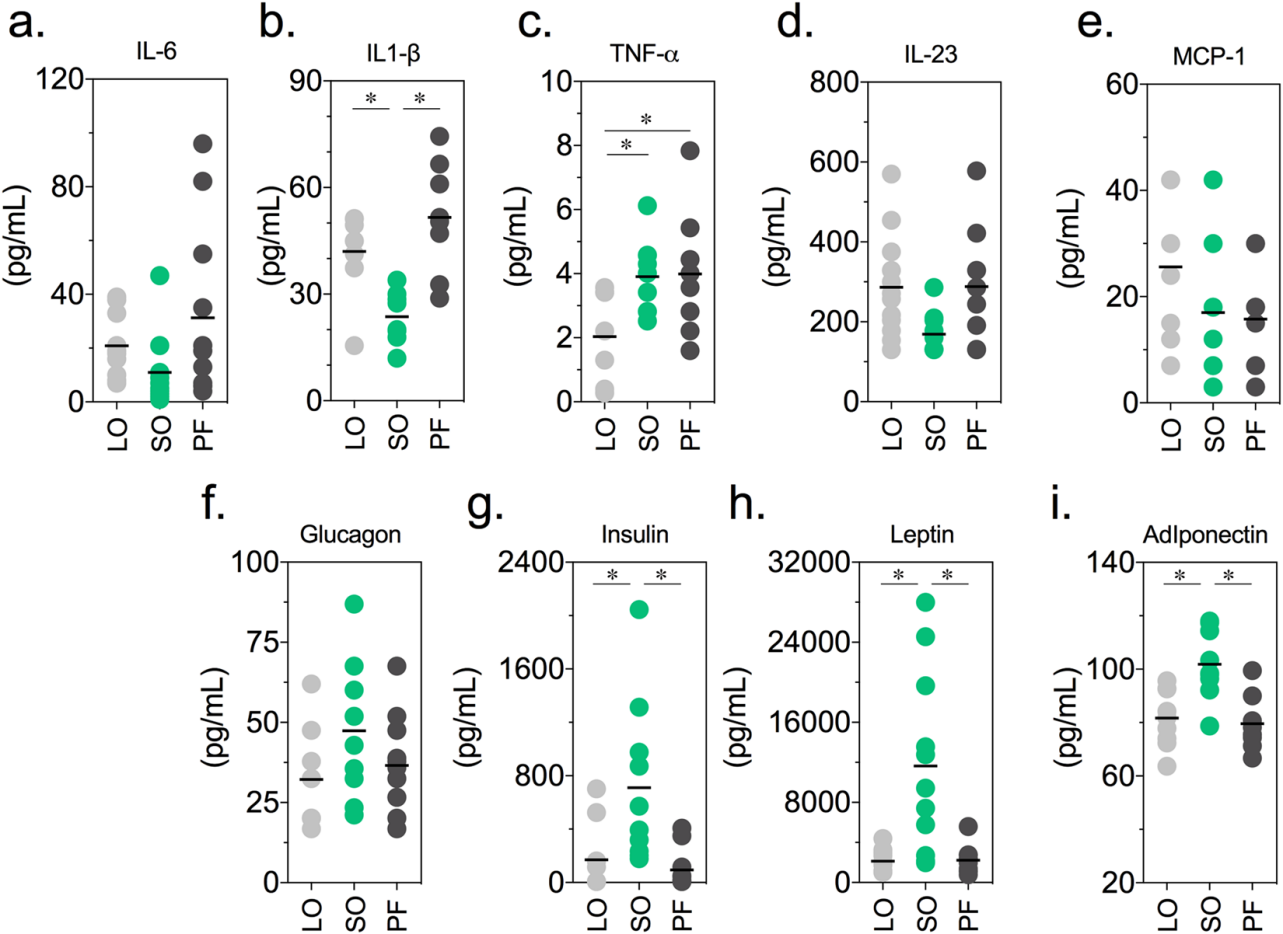
Dietary fatty acids in normolipidic diets affect result in differential profiles of serum hormones, adipokines and pro-inflammatory cytokines. C57/BL6 mice fed the specified diets for 32 weeks were sacrificed and blood was collected for the determination of serum concentrations of cytokines (IL-6, IL-1β, TNF-α IL-23, and MCP-1) and hormones (**a**: glucagon, insulin, leptin e adiponectin). n = 9; statistically significant differences are indicated by asterisks (*p* < 0.05). ANOVA and Tukey’s test.

Given the possible association between the accumulation of body fat (obesity and/or high adiposity index), a subclinical inflammation profile, increased circulating pro-inflammatory mediators and the susceptibility to different diseases, including BPH and PCa, we examined whether the above described body growth, adiposity and metabolic profiles found for the mice fed the specified diets was associated with differential profiles of circulating pro-inflammatory cytokines at the 12^th^ (Fig. S2) and 32^nd^ weeks (Fig. 3). After 12 weeks on the specified diets, no alteration was observed in the concentration of different serum hormones, cytokines and adipokines (Fig. S2).

After a longer period (32 weeks), the circulating levels of IL-6 were similar in the three groups (Fig. 3a). The animals in the PF and LO groups had elevated serum levels of IL-1β (Fig. 3b), while the LO group had reduced TNF-α compared to the other groups (Fig. 3c). IL-23 and MCP-1 remained unchanged in the three groups (Fig. 3d,e), as did the levels of glucagon (Fig. 3f). The serum concentrations of insulin and leptin (Fig. 3a) were significantly higher in the mice fed an ω-6-rich diet (SO), which showed increased body weight (Fig. 1b,c) and adiposity (Fig. 1h).

The PF diet accelerated the growth of the VP (Figs. S3b, c), increased the epithelial volume (Figs. S3d, e) and, as reported before (Escobar *et al*.^27^), promoted *Ar* expression (Fig. S3f).

### An ω-3-rich diet prevents and a saturated fatty acid-rich diet promotes enlargement of the prostate and hyperplasia of the prostate epithelium

**Figure 4:**
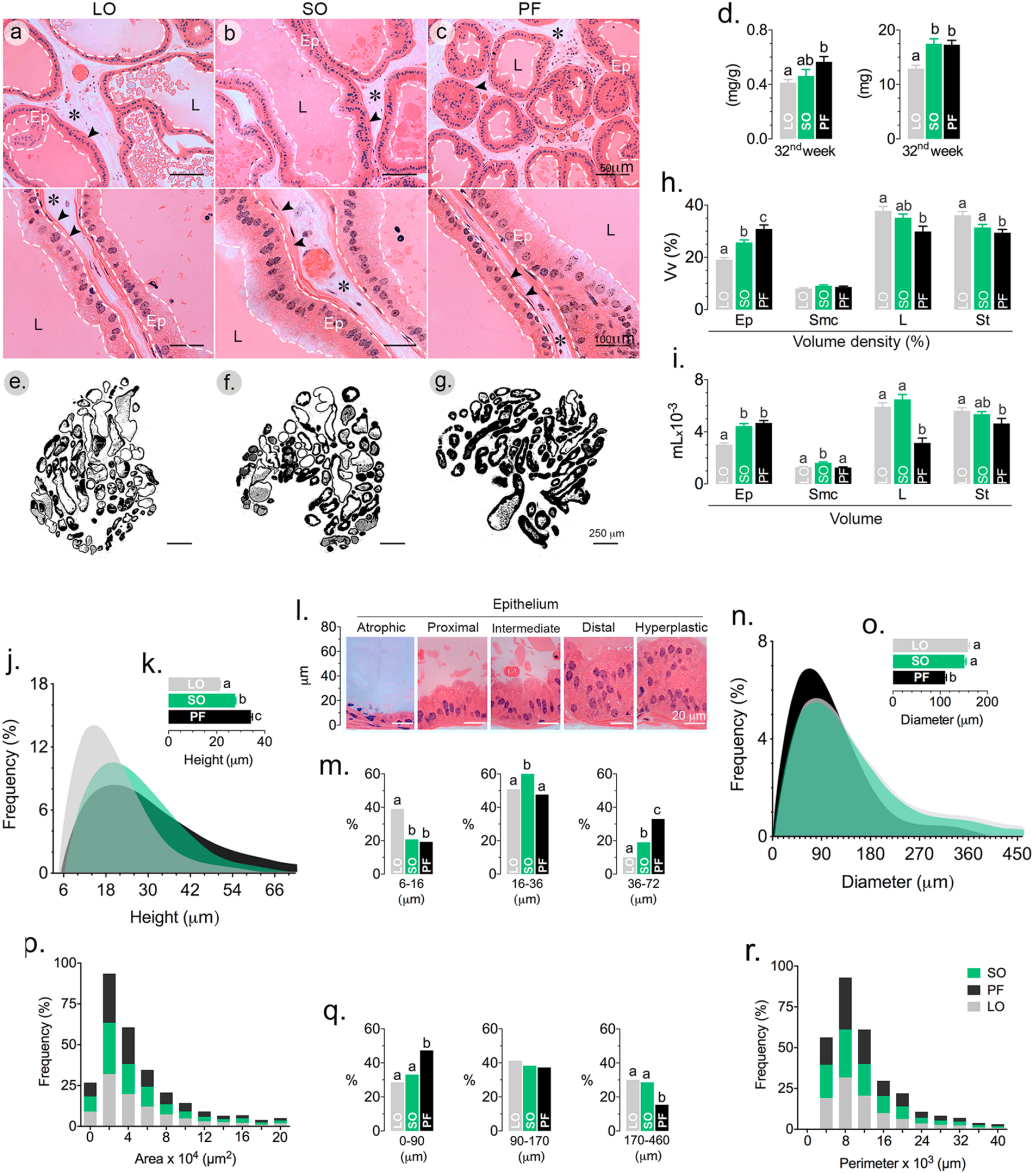
Dietary fatty acids in normolipidic diets affect results in differential prostate growth and epithelial volume. After 32 weeks on the specified diets, C57/BL6 mouse VP were dissected out, weighed and processed for routine histology (**a**–**c**,**e**–**g**). VP relative and absolute weight (**d**), volume density (V_vo_%; **h**) and volume (**i**) of the epithelium (Ep), lumen (L), stroma (*) and smooth muscle cells (arrowheads in **a**–**c**) were determined by stereology. Morphometry was used to determine the epithelial height (**j**, frequencies; **k**, mean values; **i**, frequencies according to epithelial height subclasses); the luminal perimeter (**n**, frequencies; **o**, mean values; **p**, frequencies according to luminal perimeter subclasses); acinar area (**q**) and perimeter (**r**). n = 6; statistically different groups are indicated by letters (*p* < 0.05). ANOVA and Tukey’s test.

The above described results showed that mice fed ω-6 polyunsaturated (SO) and saturated fatty acids (PF) presented worse metabolic parameters and increased adiposity after 32 weeks on the defined diets. The obesogenic phenotype of mice in the SO group was not associated with prostate growth. Indeed, VP size was similar in both the SO and PF groups (Fig. 4a–g). However, the prostates in the PF group showed increased epithelial volume (Fig. 4h,i), increase epithelial height (Fig. 4j–l) and reduced lumen diameter (Fig. 4n,o). Though the ω-6-rich diet (SO) stimulated PV growth (Fig. 4d) and resulted in increased epithelial volume (Fig. 4i), the volume of lumen was preserved, similar to that found in mice from the LO group. In this latter group, the prostate was smaller, the lumen was wider and the epithelium was shorter and frequently atrophic (Fig. 4l–o). No difference was found in the acinar perimeters (Fig. 4p–r**)**.

### Saturated fatty acid-rich and ω-6-rich diets show opposing effects on inflammation. Saturated fatty acid-rich and ω-6-rich diets affect the immune system and inflammation differently. Saturated fat promotes prostatitis, while ω-6 prevents it

**Figure 5:**
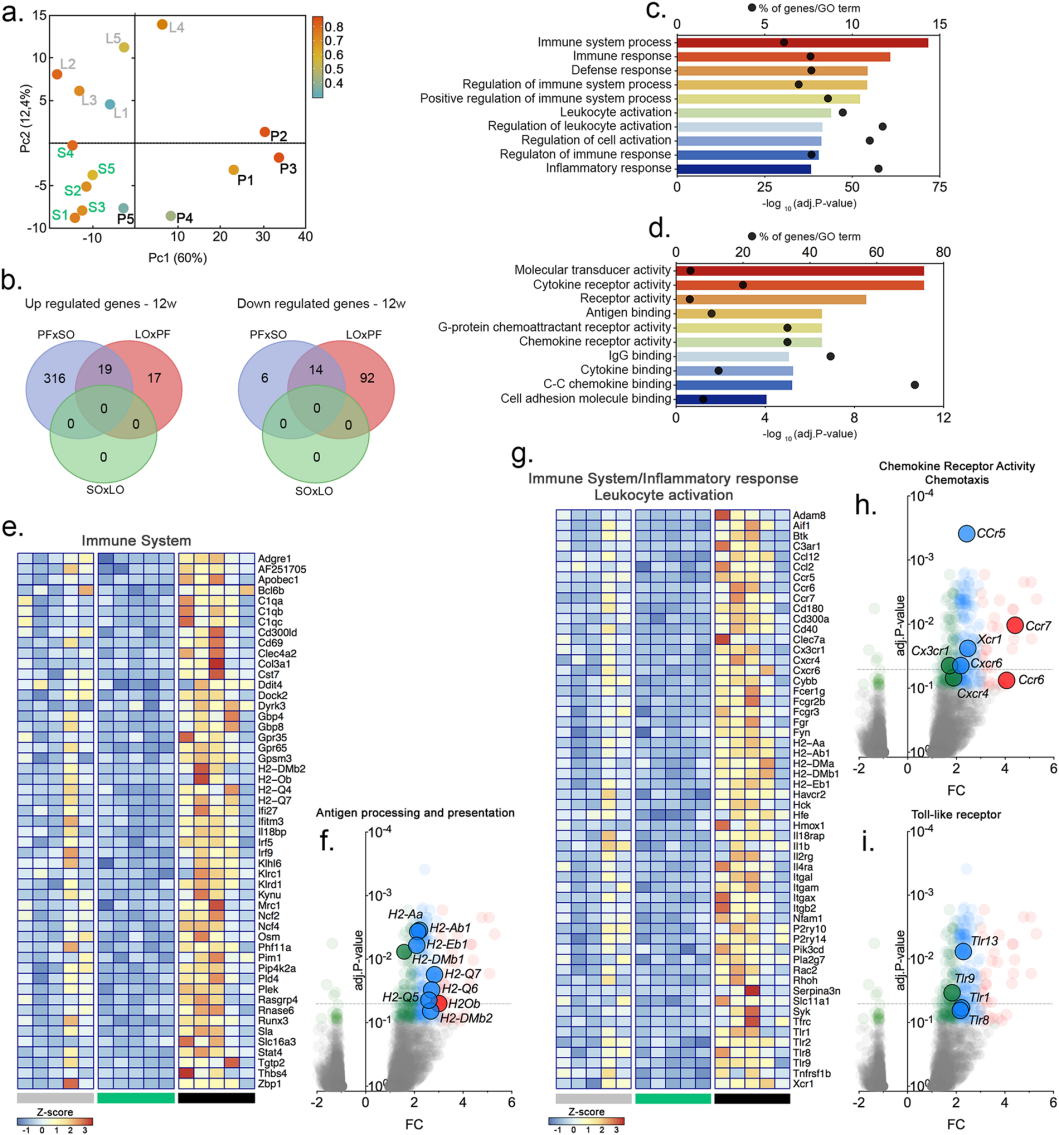
Dietary fatty acids in normolipidic diets modulate the immune system and inflammation in the mouse VP. C57/BL6 mice were fed the specified diets for 32 weeks and the VP transcriptome was examined by RNAseq. Principal component analysis (PCA) segregated the PF results from the other two groups, which were segregated by the second component. (**a**) The number of genes differentially expressed in the different groups are presented as Venn diagrams. (**b**) Biological processes and molecular functions affected by the different diets are shown in (**c**,**d**) according to their significance; bars represent the p-values and dots represent the ratios between the number of genes differentially expressed and the total number of annotated genes (enrichment) within each ontology. Genes differentially expressed were represented in Volcano plots in a pair-wise format; the graphs show each gene’s expression (Log2 fold change) and the corresponding p-value for each pair-wise comparison. Genes related to protein localization/targeting to the EA, ER stress and fatty acid biosynthesis were also represented in a heatmap (**e**–**i**), using normalized expression values (Z-Score) for each gene (n = 5).

Mice fed on the specified diets for 12 weeks showed distinct prostate and epithelial growth and different aspects of liver histology (Fig. S1). To further characterize the inflammatory profiles of the VP from animals in the different experimental groups, we evaluated the inflammatory signatures in each condition using RNAseq. The three experimental groups segregated quite well in principal component analyses (PCA). PF was separated from the other two groups in the first component, while the other two groups segregated in the second component (Fig. 5a).

A total of 355 genes were found differentially expressed in the VP from mice in the PF group, compared to the SO group, including 335 up-regulated genes and 20 down-regulated genes (Fig. 5b). Comparing the PF and LO groups, we found 142 genes differentially expressed (36 genes were up-regulated and 106 were down-regulated). No genes appeared differentially expressed when the LO and SO groups were compared to each other.

Pathways, networks and biological process enrichment was calculated for gene groups using the Metacore software (Thomson-Reuter, Boston MA, USA). Comparison between the PF and SO groups revealed 1,822 biological processes, 97 pathways and 27 networks that were significantly enriched (*p* < 0.05). Most of the biological processes were related to the immune system and inflammatory response, as well was their regulation (Fig. 5c,d). Networks were related to chemotaxis, leukocyte chemotaxy, IL-4-mediated inflammation and the JAK/STAT pathway, antigen presentation, phagocytosis, NK cell activity and lymphocyte proliferation. Finally, pathways were related to the Toll-like receptors (TLR1, TLR2, TLR4, TLR8, TLR9, TLR13 IL1β and PI3K), integrin-mediated cell adhesion and migration, the chemokines CCL2, CCL16, CCL20, CXCL16 and CCL25 (including the receptors CCR5, CCR6, CXCR6 plus PI3K and Stromelysin-1), chemotaxy mediated by the chemokine-receptors CXCR4 and CCR3 (CCL8, CCL7 and CCL3), complement (C3a) activation-mediated signaling, immunological synapsis formation and oxidative stress. For the down-regulated genes, networks related to circadian rhythms and protein folding (Figs. 5e–i and S4)

When the PF and LO groups were compared to each other, 1,082 and 117 significantly enriched biological processes, respectively, while a single network related to the role of NIMA (NEKs) protein kinases in the cell cycle was found for the down-regulated genes.

### Long-term feeding of a normolipidic, saturated fatty acid-rich diet results in endoplasmic reticulum stress and activation of the unfolded protein response (UPR)

The above described results show that mice fed for 12 weeks on a saturated fatty acid-rich diet (PF) have enlarged prostates (Fig. S1), overgrown epithelia and differential expression of genes related to the immune system and inflammation (Fig. S3). These effects were not observed in animals fed the poly-unsaturated fatty acid-rich diets.

**Figure 6:**
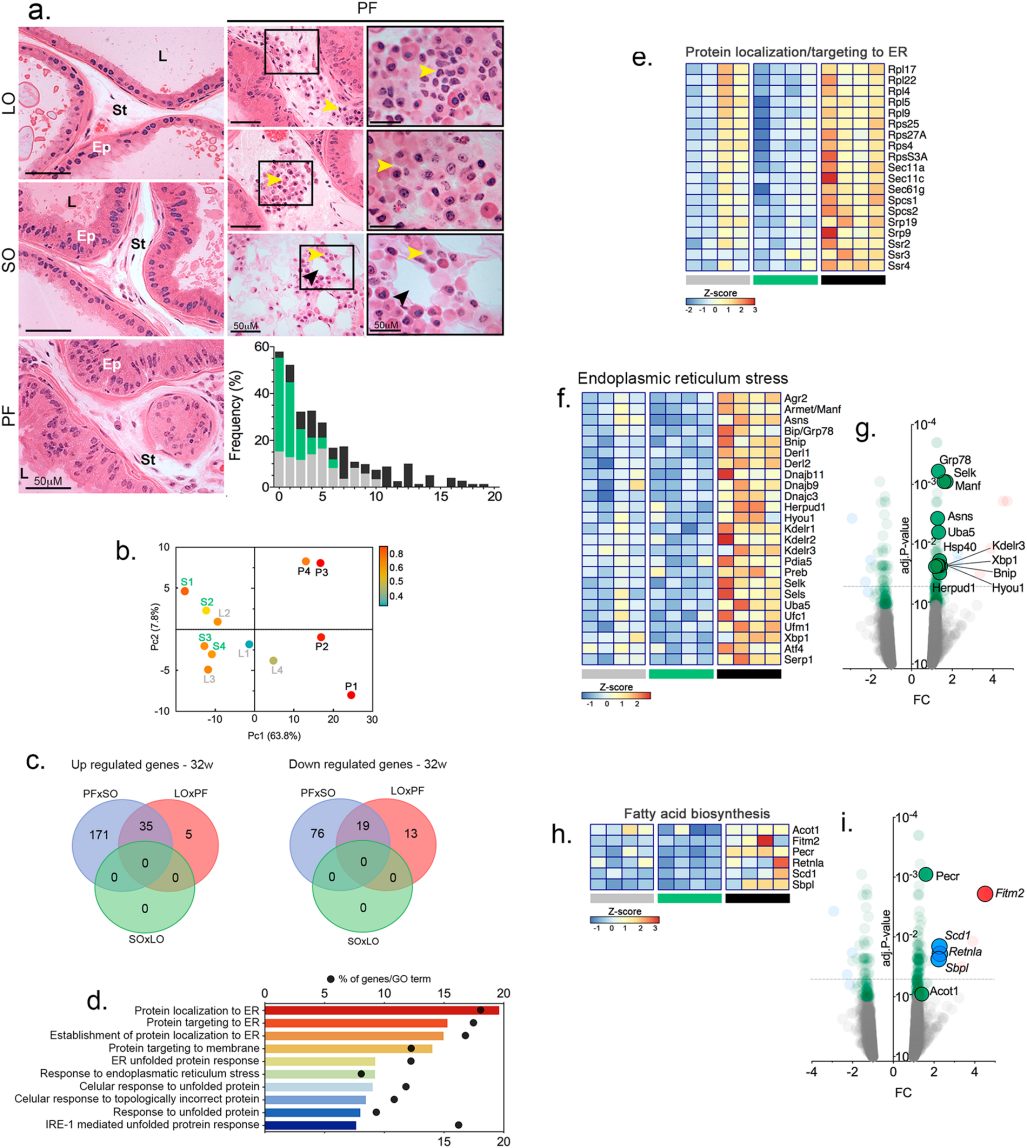
Dietary fatty acids in normolipidic diets upregulated the expression of genes related to protein folding, endoplasmic reticulum stress and the unfolded protein response (UPR). C57/BL6 mice were fed the specified diets for 32 weeks and examined for the presence of inflammatory infiltrates (**a**) and the VP transcriptome was examined by RNAseq (**b–i**). Histology revealed the presence of inflammatory infiltrates in both LO and PF groups. Yellow arrowheads point to inflammatory cells. The black arrowheads indicate an adipocyte. Ep = epithelium; St = stroma; L = lumen. The graph presents the number of inflammatory cells per histological section. Biological processes and molecular functions affected by the different diets are shown in (**b**) according to their significance; bars represent the p-values and dots represent the ratios between the number of genes differentially expressed and the total number of annotated genes (enrichment) within each ontology. Principal component analysis (PCA) segregated the PF results from the other two groups, which were not completely segregated by the second component. (**c**) The number of genes differentially expressed in the different groups are presented as Venn diagrams. (**d**) Genes differentially expressed were represented in Volcano plots in a pair-wise format; the graphs show each gene’s expression (Log2 fold change) and the corresponding p-value for each pair-wise comparison. Genes related to protein localization/targeting to the ER, ER stress and fatty acid biosynthesis were also represented by a heatmap (**e–i**), using normalized expression values (Z-Score) for each gene (n = 5).

After 32 weeks, the animals in the PF group presented epithelial hyperplasia (Fig. 3) and associated inflammatory infiltrates (Fig. 6a). In contrast, mice fed an LO diet showed frequent epithelia atrophy, often associated with the presence of intraluminal inflammatory cells.

PCA analysis of the transcriptome results unveiled that the animals of the PF group segregated with the first component, while those from the SO and LO groups were not segregated by the second component, as occurred at 12 weeks on the diets (Fig. 6b). The results also showed that 329 genes were differentially expressed in the VP of animals from the PF groups, compared to the SO group. From this total, 221 genes were up-regulated and 108 genes were down-regulated. Comparisons between the PF and LO groups revealed 72 genes differentially expressed, 40 of them up-regulated and 32 down-regulated (Fig. 6c). No differential expression was found in the comparison between the LO and SO groups.

The enrichment in pathways, networks and biological processes were calculated for groups of genes using the Metacore software, as described above. Consequently, 322 biological processes, 8 pathways and 6 networks were found to be significantly enriched (*p* < 0.05) in the comparison between the PF and SO groups, most of which were related to protein localization and ER stress, including the unfolded protein response (UPR) (Fig. 6d). Endoplasmic reticulum stress, adaptive response to stress and fatty acid biosynthesis were also enriched in the PF group when compared to the LO group, although the total number of differentially expressed genes was smaller. Genes differentially expressed are shown in Fig. 6e–i.

### Saturated fatty acid-rich (PF) and ω-3-rich (LO) diets increase the incidence of epithelial lesions in the Mongolian gerbil VP

**Figure 7:**
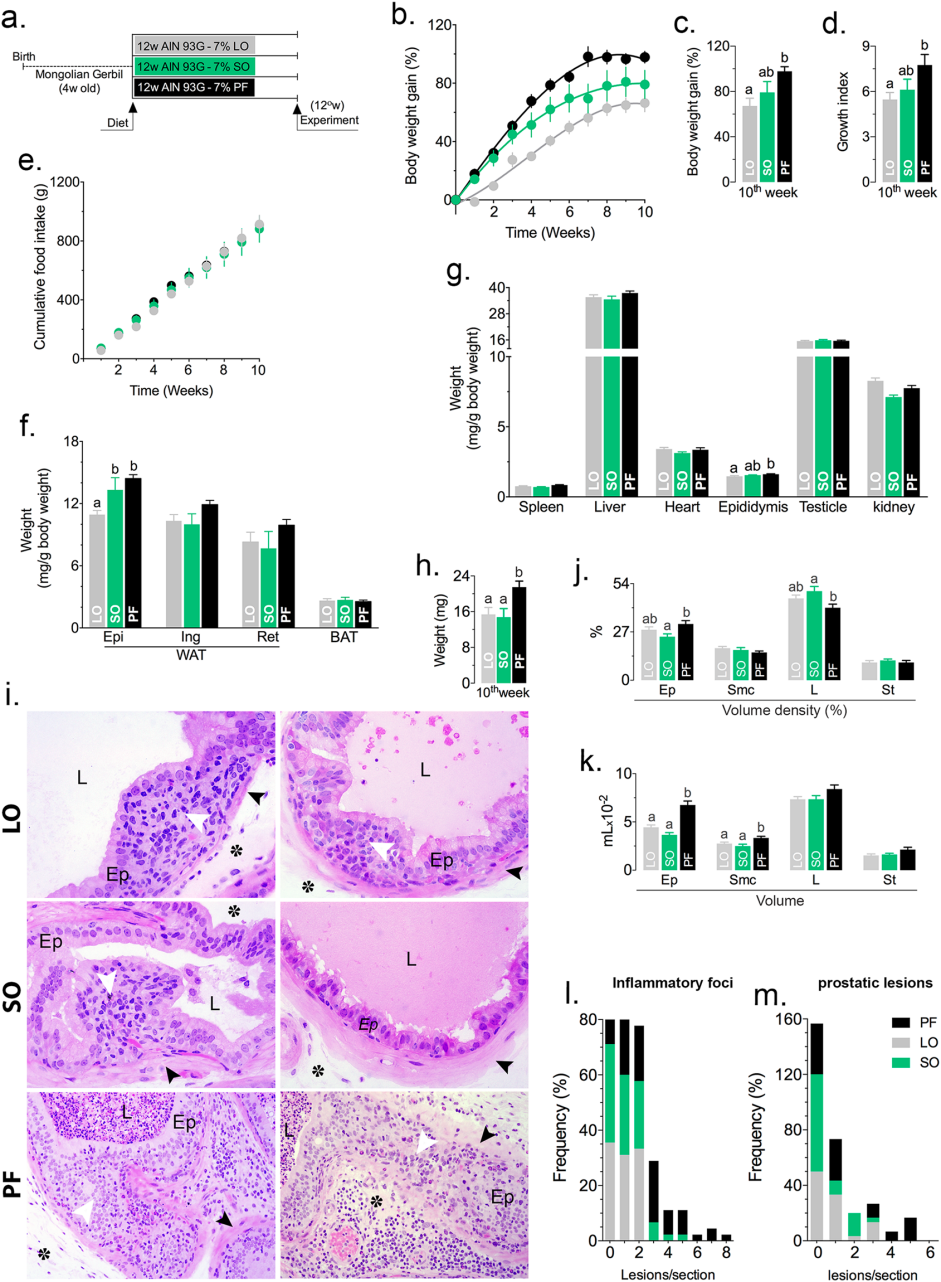
Dietary fatty acids in normolipidic diets increase the incidence of inflammation and epithelial lesions in the VP of Mongolian gerbils. Mongolian gerbils were fed the specified diets for 12 weeks. (**a**) Body weight (**b,c**), growth (**d**) and food ingestion (**e**) were measured weekly. At the end of the experiment, the animals were sacrificed and the different fat depots (**f**) and organs (**g**) were weighed and examined macroscopically. The VP was weighed (**h**), processed for routine histology and stained with HE. The histology of the VP is presented in (**i**). The volume density (V_V0%_; **j**) and volume (**k**) of the epithelium (Ep), lumen (L), stroma (*) and smooth muscle cells (arrowheads in **i**) were determined by stereology. The incidence of inflammatory infiltrates (**l**) and epithelial lesions (**m**) was determined for animals fed each diet. n = 6; statistically significant differences are indicated by letters or asterisks (*p* < 0.05). ANOVA and Tukey’s test.

The above described results showed that the ω-6-rich diet (SO) was obesogenic and that the saturated fatty acid-rich diet (PF) exhibited increased expression of inflammation-related genes and genes related to the ER stress. The LO diet showed an intermediate response associated with frequent epithelial atrophy. To extend these results to another species and to test the effects of the specified diets in an animal with a particular physiological detour that oxidizes fatty acids to produce metabolic water, Mongolian gerbils were fed for 12 weeks after weaning, according to the same protocol used for the mice (Fig. 7a). The PF diet showed an increased obesogenic potential (Fig. 7b,c): gerbils in the PF group had a higher growth rate (Fig. 7d) in spite of similar food consumption (Fig. 7e), larger epididymal fat depot (Fig. 7f) and heavier epididymis (Fig. 7g). In addition, the VPs were larger and heavier (Fig. 7h) and had increased epithelial and smooth muscle cell volumes (Fig. 7j,k). The gerbils in the PF group also had an increased number of inflammatory focci (Fig. 7l) and more frequent epithelial lesions (Fig. 7i,m). The gerbils in the LO and SO groups had smaller VPs with smaller epithelial and smooth muscle volumes. The number of inflammatory focci and epithelial lesions were also smaller, compared to those fed a PF diet.

## Discussion

In this study, we used normolipidic, isocaloric (Table S1) diets containing 7% linseed oil (67.4% PUFA, ω3/ω6 = 3.70), soybean oil (52.7% PUFA, ω3/ω6 = 0.1) or lard (13.1% PUFA, ω3/ω6 = 0.07) to feed mice for 12 or 32 weeks and Mongolian gerbils for 12 weeks and to evaluated systemic parameters and their impact on ventral prostate (VP) morphology, gene expression and inflammation.

The PUFA-rich diet prepared with LO and containing a high ω3/ω6 ratio resulted in smaller and leaner mice with healthy livers and healthy glucose metabolism. Surprisingly, the SO group, fed diets containing high contents of PUFA and a low ω3/ω6 ratio, showed the opposite effect, resulting in heavier mice, increased body gain, elevated adiposity and hepatic steatosis. These results are consistent with previous studies that show the consumption of ω-3-rich fatty acids prevents obesity, insulin resistance and hepatic steatosis, features of the metabolic syndrome^29–31^.

Importantly, mice in the PF group exhibited a biphasic growth pattern. Initially, the body growth rate was similar to that presented by the SO group. A marked transition appeared around 10–12 weeks after birth, when the PF-fed mice adopted a growth rate similar to the LO group. Only the epididymal fat was larger in the PF mice, compared to the LO group.

The obesogenic potential of SO was demonstrated in many studies using a high fat diet^30,32–34^. However, this is the first study to show the obesogenic effect of SO in normolipidic diets (7% fat). The growth patterns showed a tight and inverse association with the thermogenic capacity of the animals in the three experimental groups. Mice in the SO group had reduced metabolism and decreased thermogenesis, in contrast to the other groups. The higher thermogenesis presented by animals in the LO and PF groups was associated with a higher content and increased mitochondrial content, which also demonstrated an increased content of UCP-1, in the interscapular brown adipose tissue (BAT) and in the subcutaneous white adipose tissue (eWAT).

Arachidonic acid-derived metabolic products inhibit mitochondrial biogenesis and UCP1 expression/synthesis in the interscapular brown adipose tissue, as well as the browning of the subcutaneous white adipose tissue^33,35^. Thus, it is expected that ω-6 fatty acids present in SO might contribute to the production of arachidonic acid, which in turn inhibits thermogenesis. On the other hand, the predominance of ω-3 fatty acids in LO contribute to the synthesis of UCP1 and browning of the eWAT, thereby affecting caloric expenditure and thermogenesis, reducing fat depots and decreasing animal growth^35–37^. The increased concentrations of serum leptin, and insulin in mice from the SO group, along with the increased adiposity and glucose intolerance, represent a compensatory mechanism to reduce food ingestion and stimulate caloric expenditure^37^. The increased adiponectin seems out of context, as it was expected to decrease.

Obesity has a central role in the onset and progression of different diseases. Secretion of a variety of adipose-tissue derived factors influences other tissues, overall metabolism and susceptibility to diseases. Accordingly, obesity and insulin resistance are risk factor for BPH and PCa.

The physiological and metabolic parameters resulting from the exposure to normolipidic, isocaloric diets containing different fatty acid profiles allowed the dissociation of adiposity (i.e. total volume and adipocyte hypertrophy) and prostate growth, given that SO and PF resulted in different adiposity parameters, but similar VP growth. Furthermore, PF resulted in less adiposity, but produced perturbed VP structures and increased epithelial volumes. The results show that PF causes prostate epithelial hyperplasia, while SO causes prostate enlargement (i.e., preserved proportions among different tissue compartments).

Additionally, mice from the PF or LO groups presented similarly sized fat depots, but had completely different effects on the VP. PF caused an increase in VP weight, associated with epithelial hyperplasia, while LO resulted in a smaller VP with frequent foci of epithelial atrophy. This observation suggests that adiposity alone cannot explain the effects observed in the prostate.

The VP from animals fed for 32 weeks on a lard-based diet, which were larger and showed epithelial hyperplasia, presented an increased number of inflammatory cells per microscopical field, clearly representing inflammatory infiltrates. This is an important finding because it establishes a direct relationship between the diet and aseptic (non-infectious) prostatitis, which has been an enigma in prostate biology. Inflammatory infiltrates were also observed in mice fed an LO diet. The nature of the inflammatory infiltrates was different from those seen in the VP of PF-fed mice. These results lead to the conclusion that both lard-based and linseed-oil based diets result in prostatitis, contradicting the idea that that ω-3 has a predominant anti-inflammatory action. In contrast, the VP in mice of the SO group presented no inflammation.

Inflammation is a common finding in BPH and PCa. The transcriptome of the VP after 12 weeks on the specified diets revealed changes in pathways and gene networks related to the immune system and inflammation, depending on the dietary fatty acid profile. PF resulted in differential expression of Toll-like receptor family members (TLR1, TLR2, TLR4, TLR8, TLR9 and TLR13). This result is consistent with the fact that these receptors, in particular TLR4, are key activators of the NFκB pathway in response to saturated fatty acids in the hypothalamus^31,38^, among other organs (Liu *et al*.^31^), including the prostate gland^39^, in models of high fat diets. Though the pro- and anti-inflammatory potential of ω-6 and ω-3 fatty acids, respectively, is widely disseminated^31,40^, we showed that the SO diet, containing a low ω3/ω6 ratio, had no detectable VP inflammation. Furthermore, the same notion predicts that the LO diet, containing a high ω3/ω6 ratio would have an anti-inflammatory action. As a matter of fact, the inflammatory gene expression profile showed by the LO mice was somewhat heterogeneous. A plausible explanation is that this diet favors the organization of the male mice into hierarchies, with resulting variation in the concentration of circulating androgens (as indicated by the high dispersion of testis weight in the LO group). The inflammatory infiltrates found in the VP in LO mice were frequently associated with epithelial atrophy.

One remarkable aspect revealed by the RNAseq is that the gene signatures demonstrated for the VP from animals fed the specified diets for 12 weeks were good predictors of the histopathology of the glands after 32 weeks in the corresponding groups. This suggests that prostatitis results from a long term adaptation of the organ to different factors, such as diet.

In addition, the hormone, cytokine and adipokine profiles observed after 32 weeks on the diets were not set by the 12^th^ week. This leads us to believe that the fatty acid profiles found in the diet have local effects in the gland, connecting environmental factors to the gene expression profiles and other parameters of the prostate gland. The above mentioned Toll-like receptors could be instrumental in the relationship between dietary fatty acids and gene expression in the prostate.

After 32 weeks on the specified diets, the transcriptome revealed that the VP from mice in the PF group differentially expressed genes associated with endoplasmic reticulum stress and the UPR. Endoplasmic reticulum stress might result in the production of pro-inflammatory mediators via activation of the NFκB pathway^38,41^. It is possible that the combination of the early expression of pro-inflammatory genes, systemic inflammatory cytokines and endoplasmic reticulum stress-triggered inflammation would result in overt prostatitis.

One particular weakness of the present work is the lack of analyses of the gut microbiota, which might have been selected by the different diets with probable effects on the studied systemic and prostate parameters.

Finally, we have shown that previous aspects of the rat^27^ and mouse VP (this work) in response to the specified diets are also observed in Mongolian gerbils. This animal was included in the analyses because of the metabolic detour to produce metabolic water via fatty acid oxidation. In general, the observed effects were similar to that in the rat and mouse, suggesting that the effects of diets are irrespective of using alternative pathways to use fatty acids. However, the biphasic growth pattern caused by the PF diet in mice was not evident in gerbils, and prominent epithelial lesions were evident in gerbils after 12 weeks on the LO and PF diets.

## Methods

### Ethical approval

The experimental design, including sample size and procedures, were approved by the State University of Campinas Committee on the Use of Experimental Animal, under protocol number 3346-1. All procedures followed the reccomendations of the Brazilian Council for the Control of Animal Experimentation (CONCEA).

### Animals

Male C57/BL6 mice (n = 120) and Mongolian gerbils (n = 15), acquired from the Multidisciplinary Center for Biological Investigation (CEMIB) at the State University of Campinas (UNICAMP) and from the Sao Paulo State University (UNESP), respectively, were randomized into three groups and maintained under constant temperature (22 ± 2 °C) and light/dark cycles (12 h:12 h). Animals received water and food *ad libitum*.

### Diets

Animals were fed isocaloric growth diets containing 7% fat prepared according to the American Institute of Nutrition (AIN-93G) (Reeves *et al*., 1993) for 12 (C57/BL6 mice and gerbils) or 32 (C57/BL6 mice) weeks after weaning. The lipid source in each diet was varied to achieve different contents of saturated/poly-unsaturated fatty acids (Table S1), using processed pork fat (lard), linseed oil or soybean oil. The diets were produced commercially (Pragsoluções, Jaú, SP, Brazil), divided in 100 g aliquots, frozen at −20 °C and given to the animals every other day to avoid oxidation and to preserve the lipid profile.

### Experimental procedures

Body weight gain (C57/BL6 mice and gerbils) was determined weekly. Food consumption (expressed on a weekly basis) was measured every other day. Two weeks before the end of the treatment with different diets, the animals (C57/BL6 mice) were submitted to glucose tolerance (GTT) and insulin tolerance tests (ITT), as well as to an acute cold resistance (thermogenesis) test. The interval between each test was at least 72 h. After 12 or 32 weeks, the animals were killed with excess anesthetics (xylazine and ketamine). Blood samples were collected from the brachial plexus into tubes containing anticoagulating compounds (EDTA-k3), centrifuged and stored in cryogenic tubes at -80 °C. The animals were dissected and the organs were collected, weighed and evaluated macroscopically. Tissue blocks were processed for routine histology and stained with hematoxylin-and-eosin (HE). Adipose tissues (interscapular, BAT) and inguinal (iWAT) were collected and subjected to immunohistochemistry or examined under the microscope for fluorescence-lifetime imaging microscopy (FLIM). The ventral prostates were collected, weighed and fixed for histology/stereology (n = 5) or snap frozen in liquid N_2_ and stored at -80 °C for the biochemical and molecular analysis (n = 5).

### Glucose tolerance test (GTT)

After 6 h of fasting, blood glucose was measured. Glucose (2 g/Kg body weight) was injected intraperitoneally. Blood glucose concentration was measured 15, 30, 60, 90 and 120 min after glucose administration using a caudal vein puncture^19^.

### Insulin tolerance test (ITT)

After 6 h of fasting, blood glucose was measured. Insulin (1 U/Kg body weight) was injected intraperitoneally. Blood glucose was measured 5, 10, 15, 20, 25 and 30 min after insulin administration using a caudal vein puncture^19^.

### Histology

The VPs were dissected out and fixed overnight in 0.45% glutaraldehyde in Millonig’s buffer pH 7.4. The liver was fixed in Karnovsky’s fixative. Fixed fragments were washed in buffer or water, dehydrated and embedded in Leica historesin. Two-μm-thick sections were cut using glass knives and HE-stained. Images were acquired using a Zeiss Axiocam 506 color camera adapted to a Zeiss AxioObserver Z1 microscope.

### Stereology

Ten microscopical fields were taken per histological section of the VP from each animal (n = 5 per experimental group). The relative volumes (volume densities) of the epithelium, smooth muscle cells, lumen and stroma were calculated using the number of points corresponding to each compartment and expressed in percentages. The volume of each compartment was determined by the product of the volume density and the mean weight of the VP, considering a density equal to 1 g/mL. Counts were performed using ImageJ software by superimposing the images onto a 165-point grid. The whole procedure was repeated as previously published^42,43^.

### Mitochondrial staining and immunofluorescence

Small fragments were cut from the BAT and iWAT and immersed in phenol red-free RPMI medium containing 200 nM mitotracker orange at 37 °C for 30 min in 24-well plates. After washing with sterile PBS, the fragments were fixed in 2% paraformaldehyde for 45 min and washed in PBS containing 0.3% Tween-20. The fragments were blocked with 4% BSA in PBS containing 0.1% Tween-20 for 4 h, then incubated with a polyclonal goat anti-UCP1 antibody (cat. SC-6529) diluted at 1:1000 (v:v) overnight at 4 °C with gentle stirring. The fragments were washed with PBS containing 0.1% Tween-20 and then incubated with a secondary antibody diluted at 1:2000 for 4 h and counterstained with DAPI. The results were observed using a Zeiss LSM780 NLO confocal microscope.

### Fluorescence-lifetime imaging microscopy (FLIM)

BAT and iWAT were dissected out, washed and maintained in PBS at 37 °C. Tissue autofluorescence was characterized using a Zeiss LSM780 NLO confocal microscope. The first parameter acquired was a spectral emission curve, and the obtained spectrum was subjected to spectral deconvolution to ascertain the major fluorescent contributors to the final autofluorescence. The fluorescence lifetime was determined using a Becker & Hickel TCSPC (Time-Correlated Single Photon Counting) system. The fluorescence lifetime curves were analyzed using the SPCImage (Becker & Hickl), according to previously published work^44,45^.

### RNA extraction, integrity and purity

Total RNA was extracted from the VP after homogenization using lysis matrix A (MP Biomedicals, Santa Ana CA, USA), containing ceramic and silica beds, lysis buffer and β-mercaptoethanol, with mechanical stirring in a FastPrep system. After homogenization and extraction, the RNA was precipitated, isolated in a silica column, digested with DNase and eluted with RNase-free water, according to the recommendations of the Illustra RNAspin Mini Kit (GE Healthcare, Buckinghamshire, UK). RNA concentration and purity was determined using a NanoVue Plus Spectrophotometer (GE Healthcare) with the absorbance at λ = 260 nm and the 260/280 and 260/280 ratios, respectively. RNA integrity was checked in a Bioanalyzer, using capillary electrophoresis, according to the recommendations of the RNA 6000 Nano kit (Agilent, Santa Clara CA, USA). Only samples with RNA integrity numbers above 8.0 were selected and used for the RNAseq procedure described below.

### Library preparation and next generation sequencing

cDNA and libraries were produced with 2 mg RNA purified from the VP samples using the TruSeq Stranded mRNA kit (Illumina, San Diego CA, USA), according to the manufacturer´s instructions. The quality of the libraries was determined using a Bioanalyzer, as described above. Each library was quantified using the KAPA Library Quantification kit and was clustered in a cBOT flow cell (Illumina). Sequencing was done using a HiSeq. 2500 platform (Illumina). Paired-end sequencing was employed, producing 100 bp (2×) sequences and a total of 1,355,974,797 fragments, of which 95% of the sequences showed scores above Q30. A mean of 120 million sequences were produced per sample, and the mean alignment with the *Mus musculus* genome was 93%. Counting of the sequences aligned with the genome was done with the HTSeqCount tool (http://www-huber.embl.de/users/anders/HTSeq/doc/overview.html). To determine the genes differentially expressed (variance analysis), the data were normalized and adjusted to a negative binomial distribution, correcting for outliers and low expression genes. For statistical analysis, the Wald test was employed and the correction for multiple tests was done using the Benjamini and Hochberg (1995) procedure. Both procedures used the DESeq. 2 tool (http://www.bioconductor.org/packages/devel/bioc/html/DESeq.2.html). Results were deposited as GSE131143, at the NCBI’s GEO.

### Western blot

VP samples were sonicated in radio immunoprecipitation assay (RIPA) buffer containing protease and phosphatase inhibitors (protease inhibitor cocktail, cat. P8340; Sigma-Aldrich, Saint Louis MO, USA), sodium orthovanadate and sodium fluoride. After a 2 h incubation, samples were centrifuged (4,000 × g at 4 °C for 10 min), supernatants were collected and protein concentrations were determined using the Bradford´s method (Bradford, 1976), using BSA as standard. Protein aliquots (30 μg) were resolved in polyacrylamide gels at 100 V for 2 h, under non-reducing conditions. After electrophoresis, the proteins were transferred to nitrocellulose membranes under controlled temperature at 400 mA for 1 h. The membranes were washed three times with TBS for 5 min and incubated in 5% BSA in PBS for 1 h. Next, the membranes were exposed to a rabbit polyclonal anti-androgen receptor antibody (cat. sc816, Santa Cruz Biotech, Santa Cruz CA, USA) diluted at 1:1000 in 3% BSA in PBS overnight at 4 °C with gentle stirring. The membranes were washed again three times in TBS-T (0.1% Tween 20 in TBS) and incubated with an Alexa Fluor 546 goat anti-rabbit Ig or an Alexa Fluor 488 goat anti-rabbit Ig, diluted at 1:3000. The images were acquired in a Typhoon 9410 scanner and analyzed using ImageQuant TL1 software. Band intensity was determined using ImageJ software.

### Cold-induced thermogenesis

After 6 h fasting, the animals were exposed to 4 °C for 5 h, between 11:00 AM and 5:00 PM, to avoid a possible circadian variation in UCP1 expression^46^. Thermal images were acquired prior to exposure and after each hour until the end of the experiment using a Testo 875 camera (Testo Ltd, Alton Hampshire, UK). Images were evaluated with Testo Thermal Image software and the mean temperature was checked at 100 points over the animal surface image (n = 5/group) for each time point.

### Data analyses and statistics

Data are presented as the mean ± standard error of the mean. When possible, individual measurements are presented along with the mean value. All quantitative data were tested for normality, and the differences among the experimental groups were tested by one-way ANOVA, followed by Tukey’s *post hoc* multicomparison test. Significance was assumed when *p* < 0.05.

## Supplementary information

Dataset 1
