# Supplementary material for: Dietary fatty acid quality affects systemic parameters and promotes prostatitis and pre-neoplastic lesions
Source: Sci Rep. 2019 Dec 17;9:19233. doi: 10.1038/s41598-019-55882-5 (PMC6917739; doi:10.1038/s41598-019-55882-5)
Supplement: Supplementary file 1 — Dataset 1 [file 41598_2019_55882_MOESM1_ESM.pdf]

SUPPLEMENTAL MATERIAL

Ferrucci, D., Silva, S.P., Rocha, A., Nascimento, L., Vieira A.S., Taboga, S.R., Mori, M., Lenz-Cesar, C. & Carvalho, H.F. (2019) Dietary fatty acid quality affects systemic parameters and promotes prostatitis and pre-neoplastic lesions.

Figures S1-S4
Tables S1 and S2

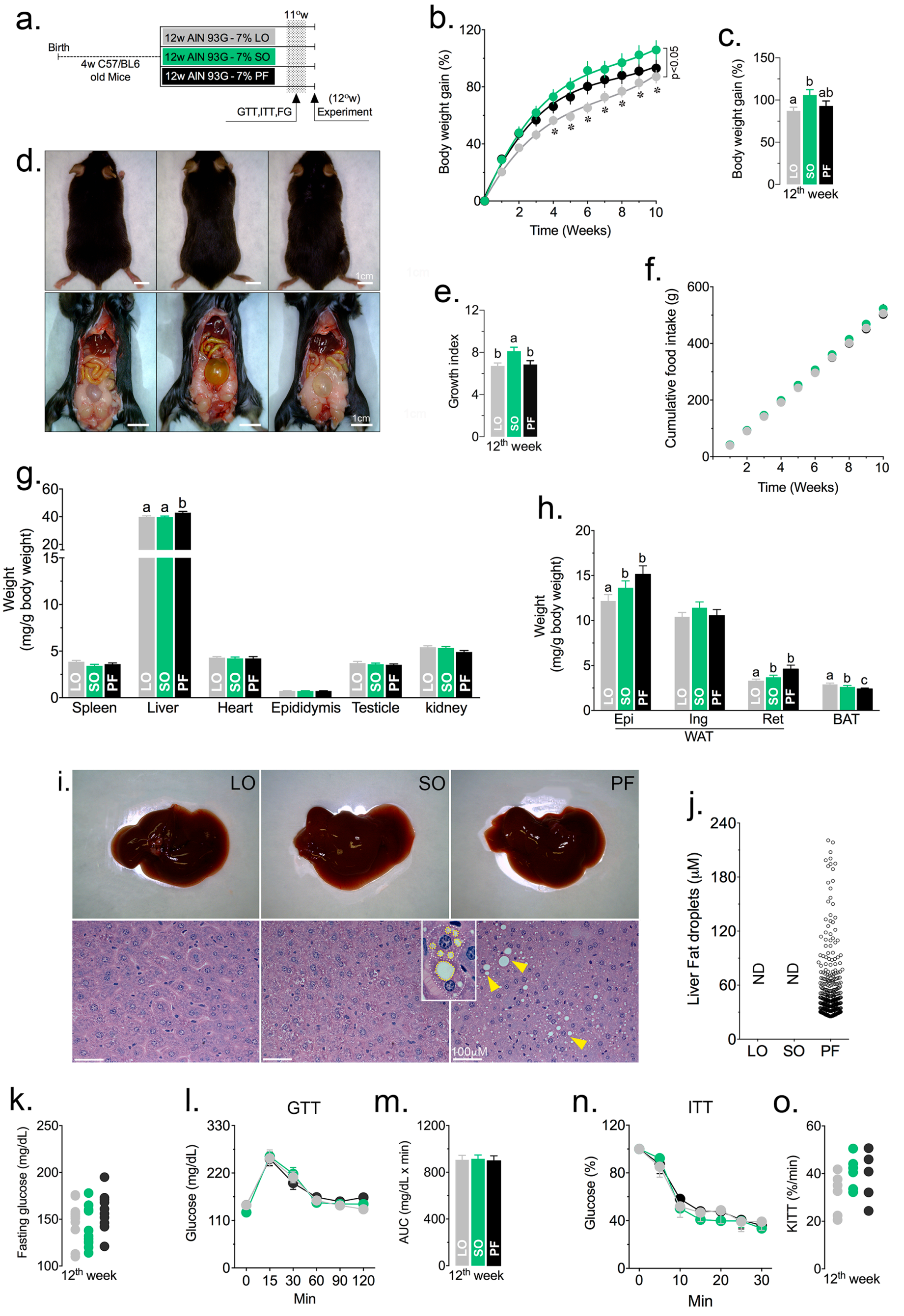

Figure S1. Phenotypical and metabolic effects of 12-week diets with different fatty acid composition. C57/BL6 mice were fed normolipidic diets for 12 weeks (**a**). Body mass gain (**b, c**), growth index (**d, e**) and food intake (**f**) were evaluated weekly. One week before the termination of the experiment (11th week) fasting glucose levels were measured (**k**) and the mice were subjected to the glucose tolerance test (**l,** mean glucose levels; **m,** area under the curve [AUC]) and the insulin tolerance test (**n,** mean plasma glucose levels; **o,** decay constant [KITT]). One week later (12th week) the animals were sacrificed and the internal organs were collected and weighed (**f**). The livers showed macroscopic alterations and were processed for histology and HE staining (**i, j**). The arrowheads indicate lipid accumulation in the the liver of PF fed animal. n = 12 (**a–h**) or n = 6 (**i–o**); statistically significant differences are indicated by letters or asterisks ($p < 0.05$).

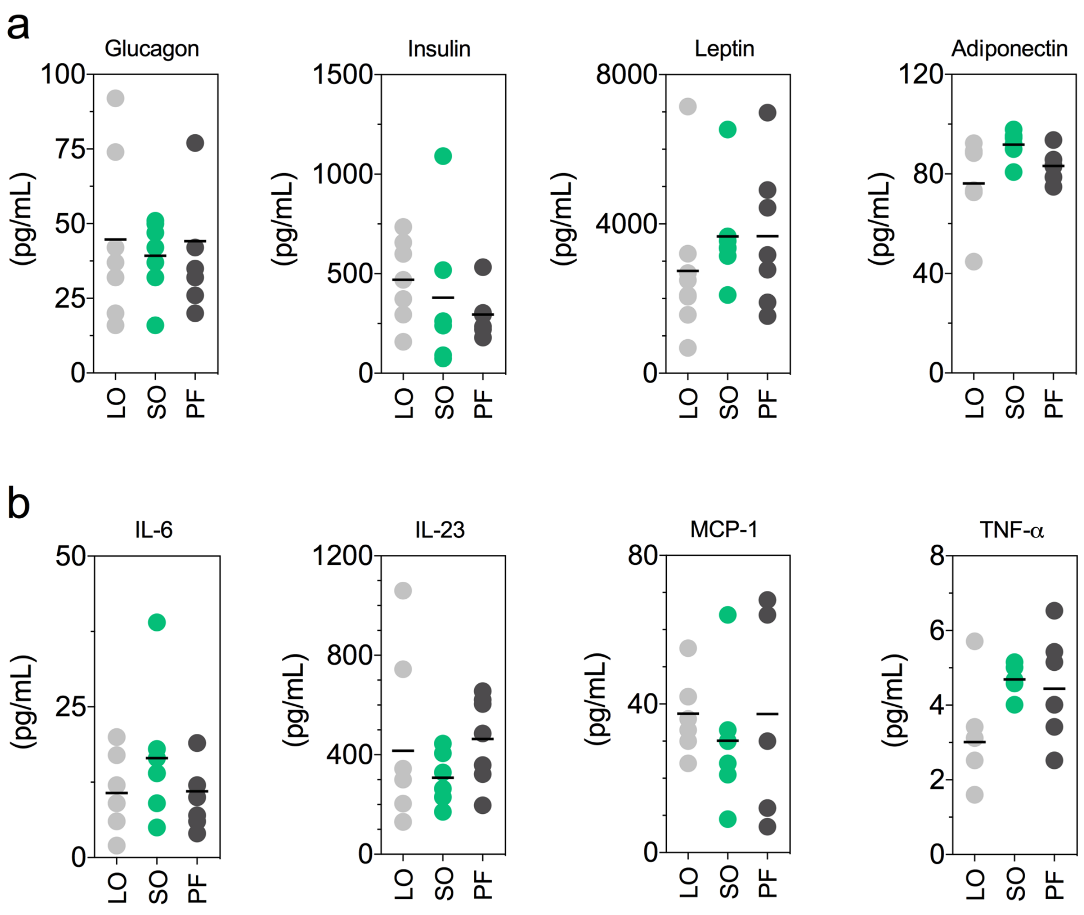

Figure S2. No changes in serum hormone, cytokine and adipokine concentrations were observed after 12 weeks on the specified diets. C57/BL6 mice were fed normolipidic, isocaloric diets prepared with different sources of fatty acids. Blood samples were collected and subjected to quantification of hormones, adipokines (**a**) and inflammatory cytokines (**b**). At this time point, the diets promoted no change in the specified signaling molecules. ANOVA was applied for statistical analysis n = 7.

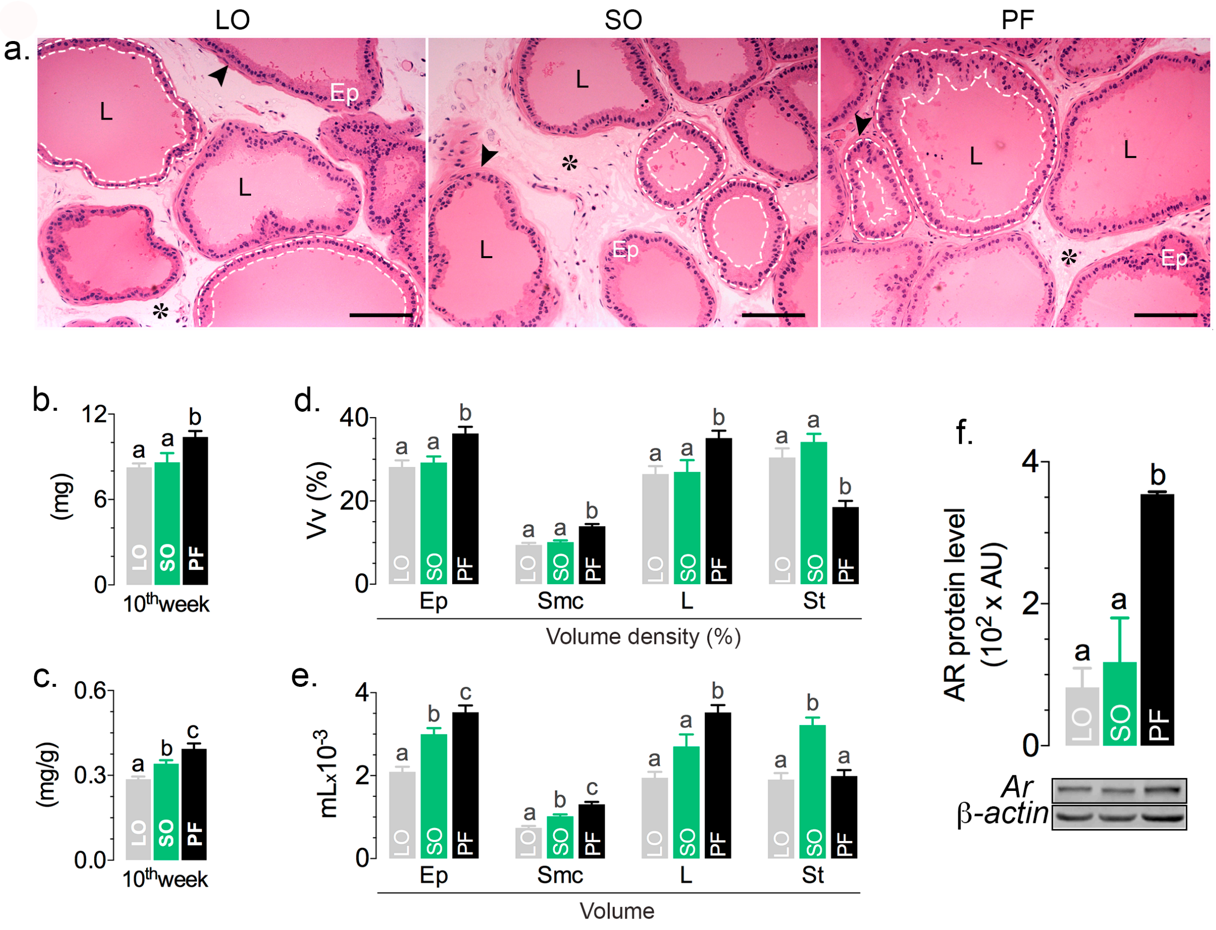

Figure S3. Dietary fatty acids in normolipidic, isocaloric diets affect prostate gland growth, histology and other parameters, such as androgen receptor expression, as earlier as 12 weeks after weaning. C57/BL6 mice were fed for 12 weeks on the specified diets. At the end of the experiment, blood was collected, and the VP was dissected out and weighed before being processed for histology and HE staining. Ep = epithelium, L = lumen, stroma (*) and smooth muscle cells (arrowheads). The saturated fatty acid-rich diet (PF) promoted increased weight (**b**) and relative weight (**c**). The histology of the glands showed normal structure, while stereology showed that the VP of animals from the PF group had increased epithelial volume, increased smooth muscle cell volume and larger lumens (**d, e**). The stromal volume was larger in animals from the SO group (**d, e**). The abundance of the androgen receptor protein was evaluated by Western blot, which showed a significant increase in animals from the PF group (**f**). The results for AR and β-actin were cropped from the same gel/blot after reacting simultaneously with both antibodies and the same exposure. n = 6 (**a–e**) or n = 5 (**f**); statistically significant differences are indicated by letters (p < 0.05).

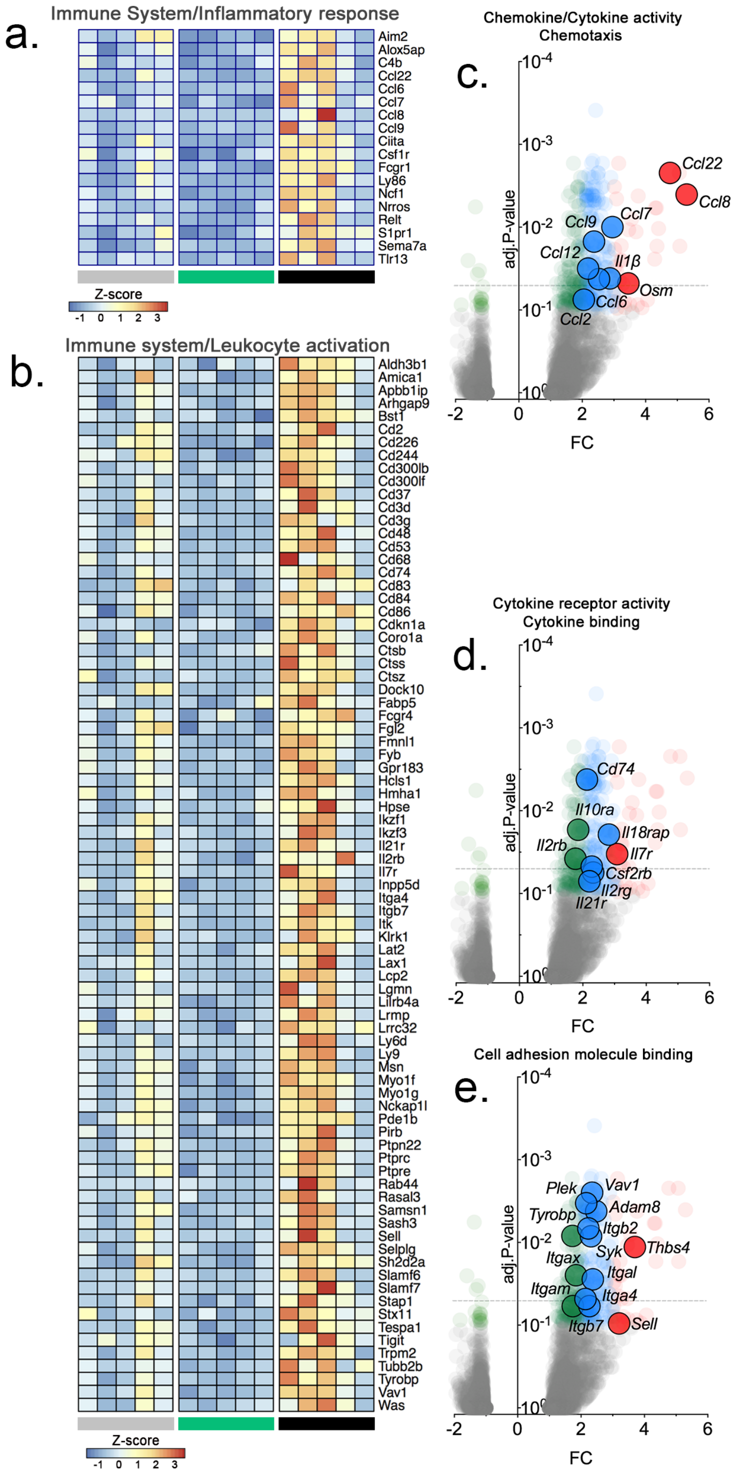

Figure S4. Dietary fatty acids in normolipidic diets modulate the immune system and inflammation in the mouse VP. C57/BL6 mice were fed the specified diets for 32 weeks and the VP transcriptome was examined by RNAseq. Genes differentially expressed were represented in Volcano plots in a pair-wise format; the graphs show each gene's expression (Log2 fold change) and the corresponding p-value for each pair-wise comparison. Genes related to protein localization/targeting to the EA, ER stress and fatty acid biosynthesis were also represented in a heatmap (**a-e**), using normalized expression values (Z-Score) for each gene (n = 5).

Table S1 . Physico-chemical composition of AIN93-G diets

Componentes	LO	SO	PF
Total	**100g**	**100g**	**100g**
Lipids	7.22 ± 0.05	7.25 ± 0.11	7.03 ± 0.14
Carbohydrates	65.78 ± 0.09	65.71 ± 0.10	65.69 ± 0.08
Proteins	15.48 ± 0.40	15.62 ± 0.14	15.85 ± 0.03
Calories	390.0 ± 0.07	391.0 ± 0.07	389.0 ± 0.09
Humidity	7.55 ± 0.15	7.51 ± 0.10	7.46 ± 0.18
Ashess	3.97 ± 0.07	3.91 ± 0.12	3.97 ± 0.09

SO, Soybean oil; LO, Linseed oil; PF, Lard.

Table S2 . Fatty acid composition of the lipid sources employed in the experimental diets, as determined by gas chromatography.

**Fatty acids**	**LO (%)**	**SO (%)**	**PF (%)**
C6:0	0.06	0.07	0.07
C8:0	0.05	0.05	0.06
C10:0	0.32	0.32	0.34
C12:0	0.12	0.14	0.22
C14:0	0.48	0.54	1.89
C15:0	0.12	0.09	0.15
C16:0	8.41	12.18	26.51
C17:0	0.18	0.16	0.52
C18:0	4.24	5.33	14.84
C20:0	0.15	0.48	0.22
C22:0	0.17	0.51	0.05
C24:0	0.15	0.24	0.07
**Σ SFA**	**14.45**	**20.11**	**44.94**
C16:1 ω7	0.25	0.25	2.31
C17:1 ω9	0.06	0.06	0.43
C18:1 ω9	17.27	26.7	38.38
C18:1 trans ω9	0.45	-	0.23
C20:1 ω9	0.15	0.21	0.6
**Σ MUFA**	**18.18**	**27.22**	**41.95**
C18:2 ω6	14.32	47.14	12.22
C18:2 trans ω6	0.07	0.47	-
C18:3 ω3	52.71	4.32	0.81
C18:3 trans ω3	0.26	0.74	0.07
**Σ PUFA**	**67.36**	**52.67**	**13.1**
**Σ ω6**	**14.39**	**47.61**	**12.22**
**Σ ω3**	**52.97**	**5.06**	**0.88**
**ω3/ω6 ratio**	**3.7:1**	**0.11:1**	**0.07:1**

SO: Soybean oil; LO: Linseed oil; PF: Lard.
